# Obstructive Nephropathy from Misplaced Suprapubic Catheter with Antegrade Migration into the Urethra

**DOI:** 10.5811/cpcem.35602

**Published:** 2025-01-19

**Authors:** Alexander W. Lipinski, Jordan R. Pollock, Nelly Tan, Douglas Rappaport

**Affiliations:** * Mayo Clinic Alix School of Medicine, Mayo Clinic, Scottsdale, Arizona; † Mayo Clinic, Department of Radiology, Phoenix, Arizona; ‡ Mayo Clinic, Department of Emergency Medicine, Phoenix, Arizona

**Keywords:** suprapubic catheter, urethral malposition, neurogenic bladder, emergency medicine, obstructive nephropathy

## Abstract

**Case Presentation:**

An 83-year-old male with a history of prostate cancer and prior prostatectomy presented with lower abdominal pain, urethral leakage, and hematuria after a routine suprapubic catheter exchange, which was found to be incorrectly positioned in the bulbar urethra, leading to obstructive nephropathy with mild hydronephrosis.

**Discussion:**

This case highlights the increased risk of suprapubic catheter misplacement and complications in elderly patients with neurogenic bladder and altered urinary anatomy, particularly after prostatectomy and artificial urethral sphincter placement. It emphasizes the importance of careful management during catheter exchanges in such patients to prevent complications of misplacement.

## CASE PRESENTATION

An 83-year-old male with a history of prostate cancer, treated with prostatectomy and artificial urethral sphincter placement, presented with severe lower abdominal pain, urethral leakage, and hematuria following a routine suprapubic catheter exchange. Pelvic computed tomography revealed that the suprapubic catheter had migrated into the bulbar urethra, resulting in malfunctioning catheter and resultant obstructive physiology with bladder distension and mild hydronephrosis (Image 1A, 1B, and 1C).

## DISCUSSION

This patient’s history presented several risk factors for complications following a routine suprapubic catheter exchange. Notably, his history of prostatectomy and artificial urethral sphincter placement suggests surgical alterations that may have caused anatomical changes, increasing the likelihood of catheter misplacement.^1^,^2^ Long-term catheterization further heightens the risk of complications such as bladder calculi, recurrent infections, granulation tissue formation, and structural changes, which can complicate future catheter exchanges.^1^,^3^ Additionally, neurogenic bladder often results in altered bladder dynamics and diminished sensation, masking symptoms of catheter misplacement and predisposing patients to bladder distension and hydronephrosis.^2^,^4^ The risk is compounded in older patients, who generally face decreased tissue elasticity, multiple comorbidities, and anatomical changes due to previous surgeries or chronic conditions.^1^,^5^

This case underscores the potential for suprapubic catheter malposition and subsequent complications in elderly patients with neurogenic bladder and altered urinary anatomy. The malpositioned catheter placement within the bulbar urethra highlights the need for heightened vigilance and specialized techniques when managing catheter exchanges in patients with altered urinary anatomy, specifically in retracting the catheter after balloon is inflated to ensure position in the bladder lumen. This report serves as a valuable reference for clinicians encountering similar cases and encourages further investigation into optimized catheterization strategies for patients with complex urological histories.

**CPC-EM Capsule**

What do we already know about this clinical entity?

*Suprapubic catheter misplacement can lead to urinary obstruction and nephropathy, especially in patients with altered urinary anatomy and neurogenic bladder*.

What is the major impact of the image(s)?

*The images clearly show a suprapubic catheter malpositioned into the bulbar urethra, causing bladder distension and mild hydronephrosis*.

How might this improve emergency medicine practice?

*Careful catheter management and vigilance for misplacement is vital, improving outcomes in patients with complex urinary anatomy*.

## Figures and Tables

**Image 1A:**
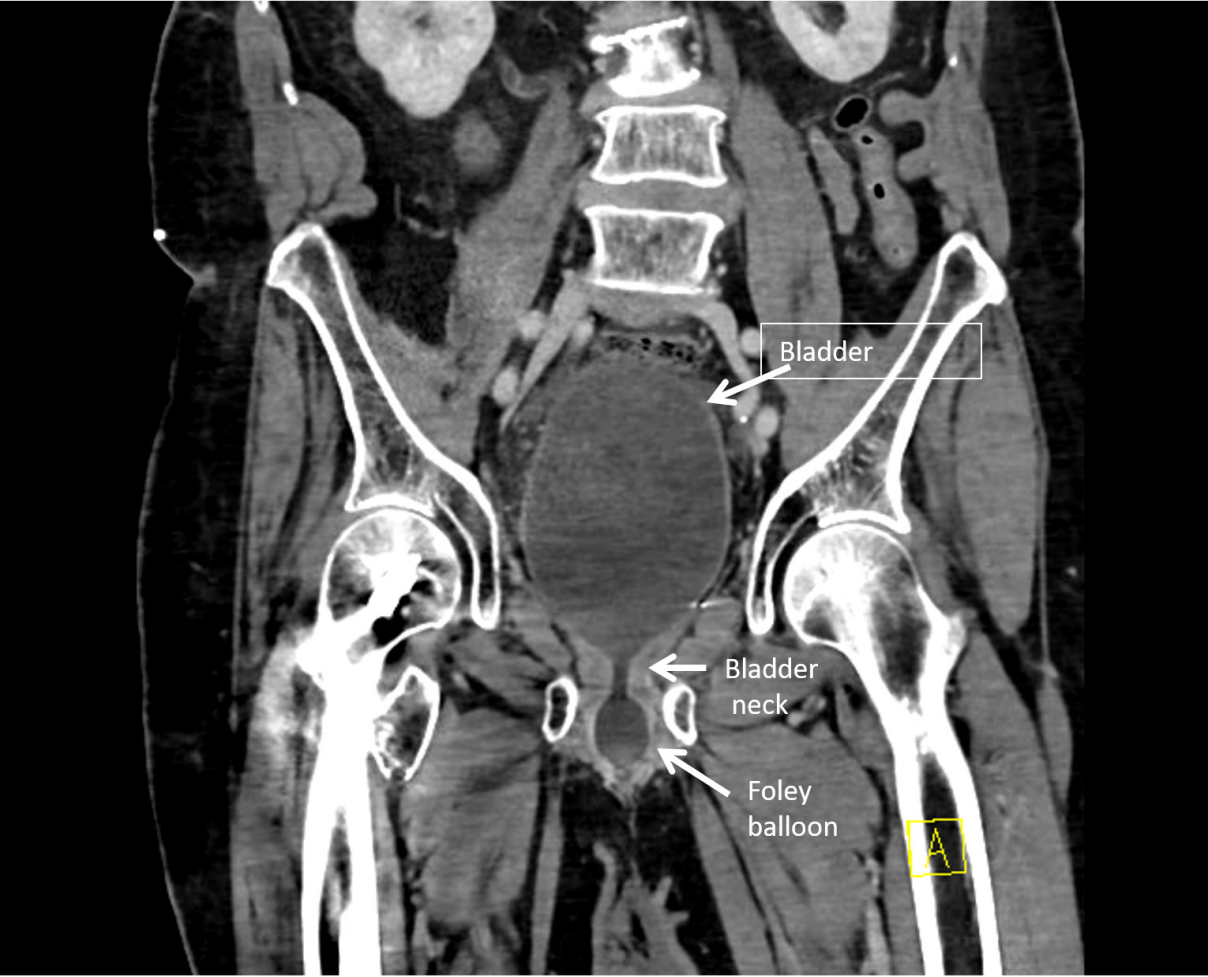
A coronal computed tomography abdomen and pelvis with intravenous contrast demonstrates a suprapubic catheter with inflated Foley balloon at the bulbar urethra caudal to the urethral anastomosis. There is also bladder distension and mild hydronephrosis from functional urinary obstruction due to malpositioned Foley balloon.

**Image 1B:**
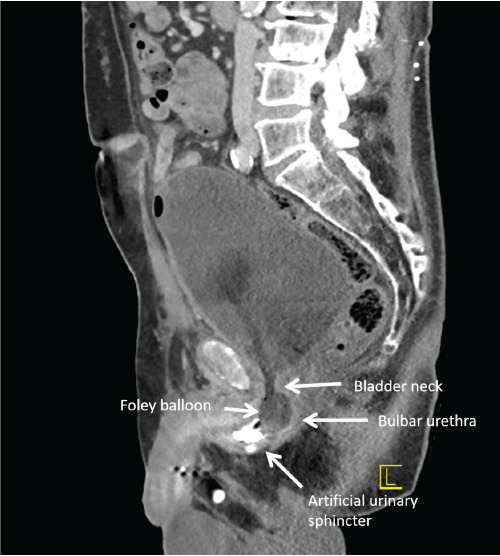
A sagittal computed tomography abdomen pelvis with intravenous contrast demonstrates a suprapubic catheter with inflated Foley balloon at the level of the bulbar urethra, just proximal to the artificial urinary sphincter. The bladder is also distended due to bladder outlet obstruction due to malpositioned catheter.

**Image 1C:**
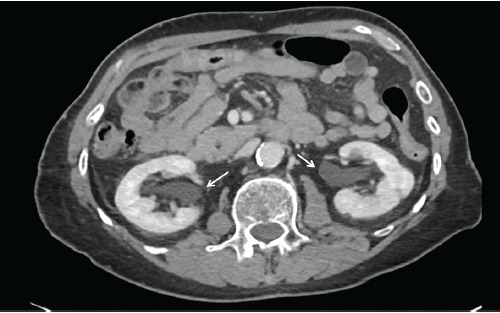
Axial computed tomography with intravenous contrast at the level of the kidneys shows mild hydronephrosis and hydroureteronephrosis from bladder outlet obstruction (white arrows).

## References

[1] Hobbs C, Howles S, Derry F (2022). Suprapubic catheterisation: a study of 1000 elective procedures. BJU Int.

[2] Li JJ, Au CF (2024). Inappropriate placement of urinary catheters into the ureter: a case report and literature review. Medicine (Baltimore).

[3] Sullivan N, AlRemeithi R, Pourmand A (2022). Suprapubic catheter encasement by bladder stone. Am J Emerg Med.

[4] Adeyemo B, Makovitch S, Foo D (2013). A peculiar complication of suprapubic catheterization: recurrent ureteral obstruction and hydronephrosis. J Spinal Cord Med.

[5] Bos BJ, van Merode NAM, Steffens MG (2022). The patient pathway for men with chronic urinary retention: treatments, complications, and consequences. Urology.

